# Highly Conductive and Long-Term Stable Phosphorene-Based Nanocomposite for Radio-Frequency Antenna Application

**DOI:** 10.3390/nano14121013

**Published:** 2024-06-12

**Authors:** Kibum Song, Seungho Ha, Keun-Young Shin

**Affiliations:** Department of Materials Science and Engineering, Soongsil University, 369 Sangdo-ro, Dongjak-gu, Seoul 06978, Republic of Korea; kibumsong121@naver.com (K.S.); hsho312@naver.com (S.H.)

**Keywords:** phosphorene, TiO_2_, polypyrrole, urea, antenna

## Abstract

In this study, an omnidirectional and high-performance free-standing monopole patch radio-frequency antenna was fabricated using a urea-functionalized phosphorene/TiO_2_/polypyrrole (UTP) nanocomposite. The UTP nanocomposite antenna was fabricated via ball milling of urea-functionalized phosphorene, chemical oxidative polymerization of the UTP nanocomposite, and mechanical pelletizing of the composite. Based on experiments, the proposed UTP nanocomposite-based antenna exhibited long-term stability in terms of electrical conductivity. After 12 weeks, a slight change in surface resistance was observed. The proposed antenna exhibited high radiation efficiency (78.2%) and low return loss (−36.6 dB). The results of this study suggest the potential of UTP nanocomposite antennas for applications in 5G technology.

## 1. Introduction

Wireless and communication technologies are becoming increasingly indispensable; consequently, the significance of the antenna, which is used to receive or transmit radio-frequency (RF) electromagnetic waves, is also increasing. Antenna characteristics, such as central operating frequency and bandwidth, are key factors in the design of RF antennas with specific bandwidth requirements. In general, the performance of an antenna is influenced by the electrical conductivity and shape of the electrode used [1].

Antenna electrodes are typically made of metallic films of Al and Cu [2]. Although these metallic materials offer the advantage of high conductivity, they are heavy, expensive, and prone to corrosion; the latter causes a decrease in their electrical conductivity [3]. Because of these limitations, efforts are being made to develop antenna electrode materials that can substitute for metallic films [4]. Among the electrode materials, phosphorene is a two-dimensional (2D) electrically conductive material composed of atomically thin layers of phosphorus that were exfoliated from bulk black phosphorous (BP) [5,6]. It has been widely utilized in memory devices, batteries, and supercapacitors owing to its large active surface area and high carrier mobility [7,8,9]. However, phosphorene has low electrical conductivity for application in antennas [10,11]. This limitation is solved by employing conductive polymers.

Many polymers typically exhibit insulating properties, whereas conductive polymers, due to their π-conjugated structures or doping, exhibit electrical conductivity. When conductive polymers are used in composites, they show the features of being lightweight and easy to fabricate [12]. Polypyrrole (PPY) is one such polymer that exhibits high conductivity and the ability to introduce functional groups, allowing it to be synthesized with other substances such as phosphorene [13,14]. However, when exposed to air or moisture, PPY oxidizes, resulting in low long-term stability of its electrical conductivity. Therefore, the use of metal oxides like TiO_2_ becomes necessary owing to their chemical stability and resistance to oxidation. Moreover, due to its excellent electrical properties, TiO_2_ is employed as a composite in electronic devices, including photocatalysts, batteries, and supercapacitors [15,16,17]. In particular, the anatase phase of TiO_2_ demonstrates superior electrical conductivity compared to other phases such as rutile and brookite [18].

Herein, we employed a simple method to synthesize a urea-functionalized phosphorene/TiO_2_/PPY (UTP) nanocomposite using mechanochemical ball milling and chemical oxidative polymerization. The synthesized UTP nanocomposite was analyzed for its material characteristics using field emission-scanning electron microscopy (FE-SEM), transmission electron microscopy (TEM), and atomic force microscopy (AFM). Additionally, Fourier-transform infrared spectroscopy (FT-IR), Raman spectroscopy, and thermogravimetric analysis (TGA) were also employed to determine the components of the UTP nanocomposite. The UTP nanocomposite was pelletized using a pellet press to be applied as an electrode to a monopole patch RF antenna. Differences in the surface resistance of the electrode based on the nanocomposite composition were investigated, and an increase in surface resistance was noted over time. Subsequently, the performance of the antenna was investigated using the nanocomposite with the lowest surface resistance, with return loss (RL) and radiation efficiency as parameters.

## 2. Experimental Section

### 2.1. Materials

Red phosphorus (RP) was purchased from Duksan Chemicals, Ansan-si, Republic of Korea. Titanium isopropoxide (TTIP, 97%), nitric acid (70%), urea (≥98%), iron (III) chloride (FeCl_3_), and pyrrole (98%) were purchased from Sigma-Aldrich, St. Louis, MO, USA. Absolute ethanol (99.9%) was purchased from Samchun Chemicals, Seoul, Republic of Korea.

### 2.2. Synthesis of Phosphorene from RP

Ball milling was used for the phase transition from RP to BP. First, 0.5 g of RP was placed in a planetary ball-milling jar along with zirconia balls (a mixture of 1 mm and 0.5 mm diameters). Milling was performed for 16 h at 500 rpm. To prevent overheating of the milling jar, the milling process was paused for 5 min after every 25 min. Next, the synthesized BP was separated from zirconia balls by sieving it through absolute ethanol. The BP solution was sonicated for 15 min and stirred for 5 min in each cycle, repeating this process three times. Following centrifugation, the obtained precipitate was dried in a vacuum oven overnight at 40 °C. Finally, the phosphorene powder was collected.

### 2.3. Synthesis of Urea-Functionalized Phosphorene (U-FP)

First, 0.3 g of phosphorene and 0.6 g of urea were placed in a planetary ball-milling jar along with zirconia balls. Milling was performed for 2 h at 500 rpm. The powder was sieved with absolute ethanol to separate U-FP from zirconia balls. Next, the ethanol solution was centrifuged to obtain the sediment. The obtained sediment was dried overnight in a vacuum oven at room temperature. Finally, the U-FP powder was collected.

### 2.4. Preparation of Anatase TiO_2_ Nanoparticles

The TTIP precursor was synthesized into anatase TiO_2_ nanoparticles using the sol-gel method. First, 0.26 mL of nitric acid was added to 200 mL of distilled water. A solution consisting of 2 mL TTIP and 18 mL absolute ethanol was added dropwise to the nitric acid solution. Subsequently, the solution was vigorously stirred for 8 h at 70 °C. After the reaction was complete, the solvent was centrifuged. The sediment was dried in a vacuum oven overnight at 50 °C. The obtained TiO_2_ powder was then heat-treated at 450 °C to synthesize the anatase phase.

### 2.5. Fabrication of Ternary UTP Nanocomposite

First, the 1 g pyrrole monomer was added to 120 mL of distilled water, and the solution was sonicated for 10 min. Next, the synthesized 0.2 g of U-FP and 0.1 g of anatase TiO_2_ nanoparticles were added to the solution and stirred for 5 min. Subsequently, a solution of 8 g of FeCl_3_ initiator and 70 mL of distilled water was added dropwise to the first solution. The resultant solution was stirred for 6 h at 5–10 °C. The synthesized UTP nanocomposites were washed with absolute ethanol and dried overnight at room temperature in a vacuum oven. The weights used in the synthesis of the UTP nanocomposite, based on 0.2 g of U-FP, 0.1 g of TiO_2_, and 1 g of pyrrole, were designated as UTP 111. Additionally, the weight ratio of TiO_2_, which increased by a factor of 9, is designated UTP 191; the weight ratio of PPY, which increased by a factor of 9, is designated UTP 119.

### 2.6. Synthesis of TiO_2_/PPY Nanocomposite

First, 1 g of pyrrole monomer was added to 120 mL of distilled water, and the solution was sonicated for 10 min. Next, 0.1 g of anatase TiO_2_ nanoparticles was added to the solution, and it was stirred for 5 min. Subsequently, a solution of 8 g of FeCl_3_ initiator and 70 mL distilled water was added dropwise to the solution. The resultant solution was stirred for 6 h at 5–10 °C. The synthesized TiO_2_/PPY nanocomposites were washed with absolute ethanol and dried overnight at room temperature in a vacuum oven.

### 2.7. Preparation of U-FP/PPY Nanocomposite

First, 1 g of pyrrole monomer was added to 120 mL of distilled water, and the solution was sonicated for 10 min. Next, 0.2 g of U-FP was added to the solution, and it was stirred for 5 min. Subsequently, a solution of 8 g of FeCl_3_ initiator and 70 mL of distilled water was added dropwise to the solution. The resultant solution was stirred for 6 h at 5–10 °C. The synthesized U-FP/PPY nanocomposites were washed with absolute ethanol and dried overnight at room temperature in a vacuum oven.

### 2.8. Fabrication of Nanocomposite-Based Monopole Patch RF Antenna Electrode

The monopole patch antenna was fabricated via the pellet-pressing process of the nanocomposite. Various nanocomposite materials were fabricated by varying the weight ratios, including U-FP/PPY and TiO_2_/PPY, to compare the long-term surface resistances of electrodes.

### 2.9. Characterization

Field emission-scanning electron microscopy (FE-SEM; Carl Zeiss GEMINISEM 300, Jena, Germany) and atomic force microscopy (AFM; System Park NX10, Santa Clara, CA, USA) were used for morphological analysis. Transmission electron microscopy (TEM) was employed using a F200X G2, Talos, MA, USA. Raman spectroscopy and Fourier-transform infrared spectroscopy (FT-IR) were performed using Renishaw Raman microscopy, Wotton-under-Edge, UK and a Bruker VERTEX70, Ettlingen, Germany, respectively. Thermogravimetric analysis (TGA) was conducted using a TGA/DSC 1 (Mettler Toledo, Greifensee, Switzerland). The surface resistance was measured using a 4-point probe; Loresta-GX MCP-T700 (Mitsubishi, Tokyo, Japan). The RL of the antennas was measured using the Keysight E8362B PNA network analyzer, Santa Rosa, CA, USA in the frequency rage 1–5 GHz. Impedance was plotted on a Smith chart by first normalizing to the characteristic impedance of the system (50 ohms). The UTP pellet-based antenna was directly connected to the subminiature version A connector without an external matching network via a feed line. As a ground plane, a rectangular copper (Cu) plate was designed to be at least 120 mm × 100 mm. The radiation pattern and efficiency of the antenna were evaluated using an anechoic chamber at the Electromagnetic Wave Technology Institute of Korea. The measuring system consists of a vector signal generator (E4438C, Agilent Technologies, Santa Clara, CA, USA), a base station simulator (E5515C, Agilent Technologies), and a wibro/wimax communication tester (CMW270, Rohde & Schwarz, Munich, Germany).

## 3. Results and Discussion

**Figure 1:**
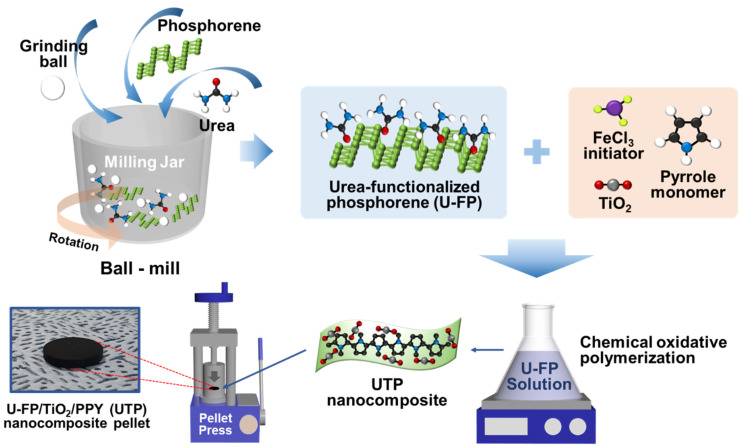
Schematic of the fabrication of UTP nanocomposite for antenna electrodes via mechanochemical ball milling, chemical oxidative polymerization, and mechanical pelletizing processes.

The UTP nanocomposite for the antenna electrode application was fabricated via mechanochemical ball milling, chemical oxidative polymerization, and mechanical pelletizing processes, as depicted in Figure 1. First, urea-functionalized phosphorene (U-FP) was synthesized via mechanochemical ball milling of phosphorene with urea. Ball milling is a suitable method for introducing urea functional groups into phosphorene nanosheets under high-temperature and high-pressure conditions.

Next, UTP nanocomposites were synthesized via chemical oxidative polymerization, which offers the advantages of bulk synthesis, a simple process, and the capability to adjust the weight ratio for the control to various properties [19]. An FeCl_3_ initiator was used to polymerize pyrrole monomers into PPY in an aqueous solution containing U-FP and TiO_2_ nanoparticles. Notably, the 2D nanosheet structure of U-FP functions as a supporting framework in polymerization. Urea in U-FP weakens the van der Waals forces between the layers of phosphorene, preventing their restacking [20]. Furthermore, the hydrogen bond formed between the hydrogen atoms of urea and the nitrogen atoms of PPY strengthens the interfacial interaction, leading to enhanced structural stability [21].

Anatase-phase TiO_2_, which is known for its excellent electrical conductivity, was synthesized through the sol-gel method from titanium isopropoxide. Finally, the synthesized UTP nanocomposite powder was pelletized using a pellet press to fabricate a free-standing antenna electrode. The diameter of the fabricated antenna was 20 mm. In the fabrication of antennas based on conductive nanocomposite, a binder is usually required. However, binders decrease the electrical conductivity of the nanocomposite. In the free-standing structure of the electrode used in this study, the absence of a binder helps maintain the electrical conductivity of the UTP nanocomposite.

**Figure 2:**
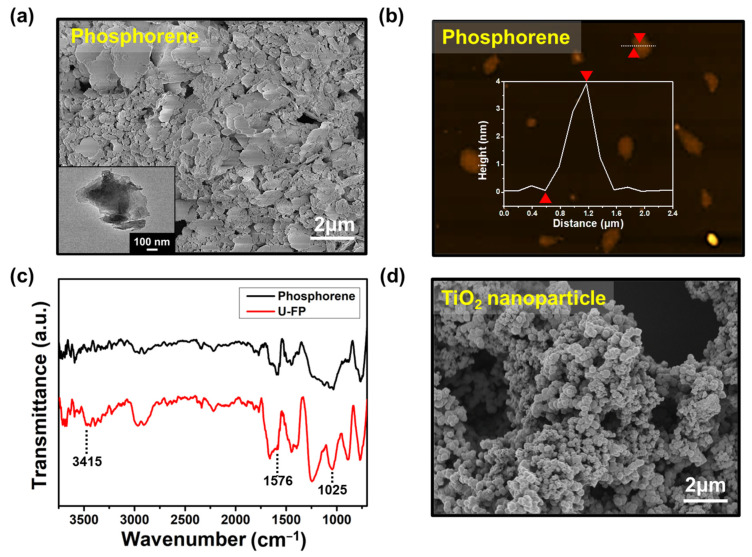
(**a**) FE-SEM and (**b**) AFM images of the 2D structure of the phosphorene nanosheet (inset in (**a**) TEM image of phosphorene). (**c**) FT-IR spectra of phosphorene and U-FP. (**d**) FE-SEM image of anatase TiO_2_ nanoparticles.

Figure 2a depicts a typical 2D structure of phosphorene, with diameters ranging from approximately 100 nm to 2 μm. The morphologies of phosphorene were examined using AFM, as depicted in Figure 2b. The image reveals phosphorene with a thickness ranging from 1 to 4 nm, indicating the successful synthesis of few-layered phosphorene. The morphologies of BP were observed using AFM, as depicted in Figure S1, revealing a stacked structure with a thickness ranging from 200 to 350 nm. The covalent bonding between phosphorene and urea can be confirmed based on Figure 2c. It shows the FT-IR spectra of phosphorene and U-FP. The broad peaks of U-FP are at 3415 and 1576 cm^−1^, which indicate N–H bonds. Furthermore, the peak at 1025 cm^−1^ indicates the presence of P–O–C bonds in U-FP [20]. It can be observed that urea functionalizes well on phosphorene. Figure 2d shows an SEM image of anatase TiO_2_ nanoparticles, with diameters ranging from 20 to 40 nm. The nanoparticles exhibit a relatively uniform size after the calcination process [22].

**Figure 3:**
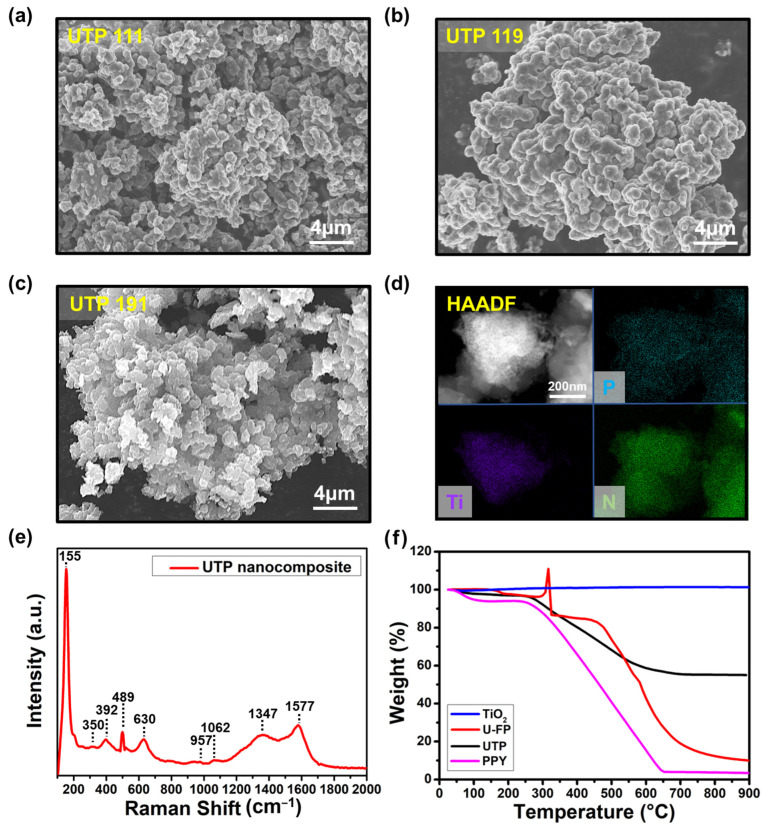
(**a**–**c**) FE-SEM image of UTP 111, UTP 119, and UTP 191. (**d**) HAADF-STEM image and EDS elemental mapping images of the UTP nanocomposite. (**e**) Raman spectra of the UTP nanocomposite. (**f**) TGA curves of the UTP nanocomposite, U-FP, TiO_2_, and PPY.

Figure 3a–c illustrates the surface morphology of PPY and TiO_2_ on the 2D structure of the phosphorene nanosheet according to the composition ratio [23]. Overall, PPY and TiO_2_ were successfully synthesized on the surface of U-FP. Moreover, U-FP is shown to serve as the framework for the synthesis of the nanocomposite. For UTP 111, it exhibited a fragment-shaped surface. Conversely, UTP 119, with the highest content of PPY, showed the smoothest morphology due to the polymerization of pyrrole. In the case of UTP 191, which has the highest content of TiO_2_, the composite exhibited a rough morphology due to the nano-sized TiO_2_ particles. Figure S2 also provides FE-SEM images for binary nanocomposites based on U-FP/PPY and TiO_2_/PPY.

The scanning transmission electron microscope (STEM) image using high-angle annular dark-field (HAADF) and energy dispersive spectroscopy (EDS) analysis of UTP nanocomposite is depicted in Figure 3d. It shows that P and N, evenly distributed in the UTP nanocomposite, indicate the formation of a nanocomposite structure with phosphorene and PPY. The presence of Ti signifies that the UTP nanocomposite contains nanocrystals of TiO_2_. Figure 3e shows the Raman spectra of the synthesized UTP nanocomposite. The peaks of TiO_2_ nanoparticles are observed at 155 and 630 cm^−1^ (indicating the E_g_ mode) and at 392 cm^−1^ (indicating the B_1g_ mode). These peaks indicate the typical tetragonal structure of anatase TiO_2_ [24]. The peaks at 350 and 489 cm^−1^ are associated with U-FP. These two peaks are attributed to the representative A_1g_ and A_2g_ peaks of phosphorene nanosheets [21]. For PPY, two broad peaks are present at 1577 and 1347 cm^−1^, attributed to the G and D bands, respectively [25]. To confirm the polymerization of PPY, the FT-IR spectrum of the UTP nanocomposite is presented in Figure S3. The peak at 1007 cm^−1^ corresponds to the P=O stretching vibration characteristic of phosphorene, while the peak at 1453 cm^−1^ represents the C=N in-plane vibration. The characteristic peaks of TiO_2_ can be identified through the Ti–O bending at 660 cm^−1^ and the Ti–OH stretching at 1636 cm^−1^. The PPY can be identified by several characteristic peaks observed in the spectrum, including N–H wagging at 783 cm^−1^, C–H out-of-plane vibration at 882 cm^−1^, a conjugated backbone peak at 1158 cm^−1^, C–H in-plane vibration at 1291 cm^−1^, and C=C stretching at 1542 cm^−1^. Based on this result, the G band indicates the presence of π-conjugated structures with double-bonded carbon, whereas the D band indicates vibrations in the aromatic ring in PPY. Furthermore, the small peaks at 1062 and 957 cm^−1^ indicate C–H in-plane deformation and C–C ring deformation. These peaks are representative characteristics of conducting polymers, indicating the presence of bipolarons and polaron structures [26]. Therefore, UTP nanocomposite was successfully synthesized via chemical oxidative polymerization.

Figure 3f shows the TGA curves of the UTP nanocomposite, U-FP, TiO_2_ nanoparticles, and PPY. It is evident that TiO_2_ retained its original weight without any loss up to 900 °C. This indicates the high thermal stability of anatase TiO_2_ nanoparticles [24]. For U-FP, there is a weight increase at approximately 290 °C because of oxygen chemisorption on its surface [27]. Subsequently, U-FP experiences a 90% weight loss at approximately 900 °C. For PPY, weight loss started at 176 °C, with a 97.5% mass loss observed at 900 °C. In this experiment, the UTP 191 sample was used for TGA analysis. For UTP 191, approximately 3.4% weight loss at approximately 258 °C and a substantial 44.6% weight loss at approximately 685 °C occurred. To compare the weight losses of individual components, namely, U-FP, TiO_2_, and PPY, with the weight loss of UTP 191, the following equation is used:

$$\mathrm{WL}\;\mathrm{of}\;\mathrm{composite}\;A_{1}A_{2}A_{3}=\frac{1}{100}\overset{3}{\underset{k=1}{\sum }}\left(\mathrm{WR}\;\mathrm{of}\;A_{k}\times \mathrm{WL}\;\mathrm{of}\;A_{k}\right)$$

where WL and WR are the weight loss and weight ratio, respectively. At approximately 258 °C, the weight losses of the three components were as follows: U-FP 3.4%, TiO_2_ 0%, and PPY 6.5%. When calculated according to their weight ratios, the result is 3.56%, which is similar to the 3.4% weight loss observed for UTP 191 at 258 °C. However, at approximately 685 °C, the weight losses for the components were as follows: U-FP 79.2%, TiO_2_ 0%, and PPY 96.2%. Upon recalculation, this resulted in 55.3% as an indication of the decreased weight loss in the UTP nanocomposite observed at approximately 685 °C. The decreased weight loss can be attributed to the various bonds among the components in the UTP nanocomposite. First, the amine groups on the U-FP surface and PPY form a hydrogen bond, which is a strong chemical bond that significantly enhances the structural stability of the UTP nanocomposite. Secondly, hydrogen bonds and electrostatic interactions exist between TiO_2_ and PPY. Hydrogen bonds are formed between oxygen atoms from TiO_2_ and hydrogen atoms from PPY, as well as electrostatic interactions between titanium atoms from TiO_2_ and carbon atoms from PPY. These interactions lead to a stable TiO_2_/PPY structure in the UTP nanocomposite [28]. These bonds and interactions enhance thermal stability and reduce the weight loss of the nanocomposite.

**Figure 4:**
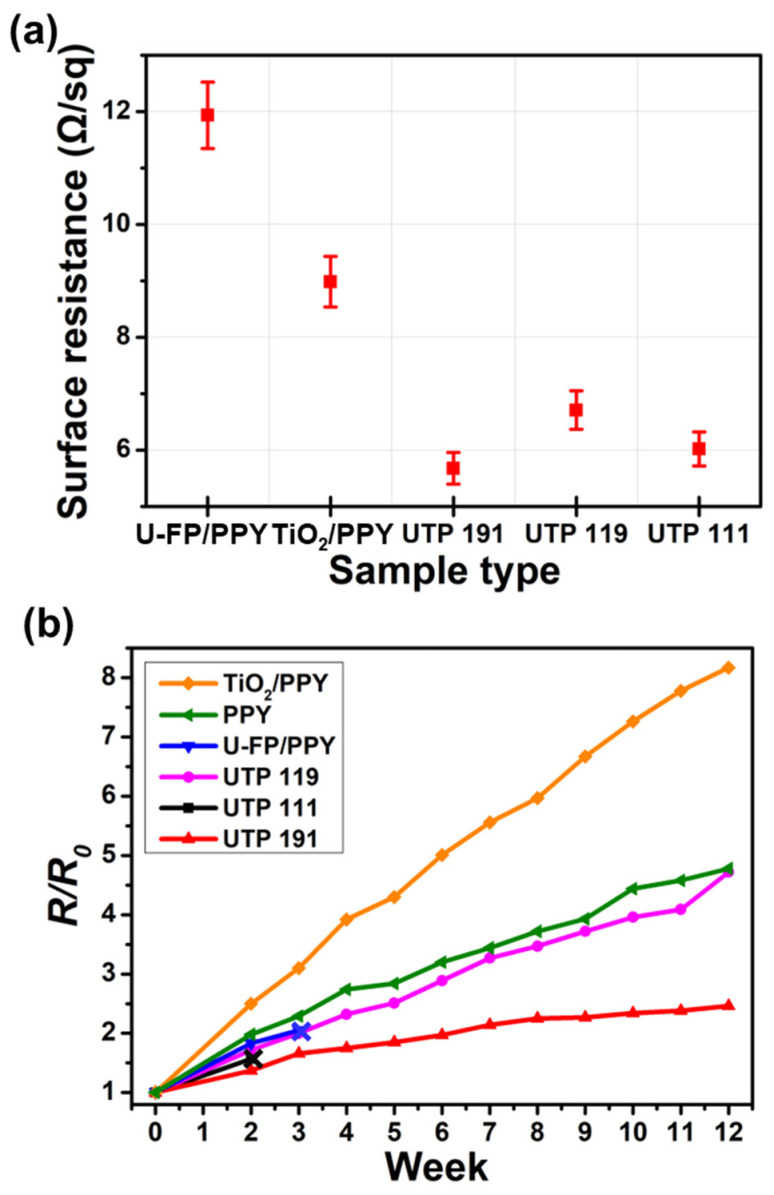
(**a**) Comparison of surface resistances of binary and ternary nanocomposite pellets with various weight ratios. (**b**) Surface resistance curves of binary and ternary nanocomposite pellets with various weight ratios, as well as of PPY, over 12 weeks. Nanocomposite pellets with a thickness ranging from 120 to 130 µm were employed.

The low surface resistance of antenna electrode materials has a significant impact on the performance of antennas. The surface resistance of binary and ternary nanocomposites with various weight ratios is depicted in Figure 4a. Binary nanocomposites such as U-FP/PPY and TiO_2_/PPY exhibit lower surface resistances compared with those of pristine U-FP and TiO_2_. The addition of PPY improves the electrical conductivity of the nanocomposites. Pristine U-FP and PPY experience a decrease in their electrical conductivities owing to oxidation. In contrast, TiO_2_ plays a role in preventing oxidation in the nanocomposite owing to its high chemical stability. Therefore, the surface resistance of TiO_2_/PPY is lower than that of U-FP/PPY. Meanwhile, ternary nanocomposites, such as UTP 111, UTP 191, and UTP 119, exhibit lower surface resistances when compared with those of binary nanocomposites. In the ternary nanocomposite, the 2D-structured U-FP serves as a beneficial framework by forming hydrogen bonds with PPY during synthesis. Furthermore, the wide specific surface areas of TiO_2_ nanoparticles enable the uniform polymerization of PPY on U-FP. Consequently, the electron channel in PPY remains uninterrupted, leading to an increase in the electrical conductivity of the nanocomposite [28]. The surface resistance among the ternary composites showed slight differences. The UTP 191 specimen, with the highest TiO_2_ content, exhibited the lowest surface resistance (5.68 Ω/sq).

Figure 4b depicts the changes in surface resistance over time. There are also binary and ternary nanocomposites with various weight ratios in PPY. A slight change in surface resistance implies an increase in long-term stability against oxidation. In general, phosphorene readily exhibits oxidation when exposed to oxygen or moisture, whereas PPY exhibits slow oxidation [29,30]. Therefore, both PPY and U-FP/PPY exhibit rapid oxidation over time. In U-FP/PPY, the pellet exhibits weak cohesion owing to a significant amount of U-FP, resulting in compromised structural stability. Consequently, the U-FP/PPY pellet easily fractures during surface resistance measurements. When TiO_2_ is added to U-FP/PPY, it exhibits low changes in surface resistance over time, attributed to its excellent structural stability and long-term resistance to oxidation. However, the presence of a significant amount of U-FP in UTP 111 compromises its structural stability. As a result, UTP 111 easily fractures during surface resistance measurements. Therefore, it is observed that UTP 191, which has the highest TiO_2_ content, exhibits a higher long-term stability against oxidation than UTP 119, which has the highest PPY content. After 12 weeks, the surface resistance of UTP 191 had slightly increased, with a resistance change ratio of approximately 2. In the case of the TiO_2_/PPY nanocomposite, despite the high content of TiO_2_, the absence of a U-FP framework hindered the fabrication of the nanocomposite. As a result, significant oxidation was observed over time since TiO_2_ failed to fulfill its intended function. As a conclusion, the presence of TiO_2_ within the nanocomposite contributes to its enhanced structural stability and increased electrical conductivity, leading to its long-term stability against oxidation. Furthermore, the UTP nanocomposite pellet can be used as an antenna because of its superior conductivity.

**Figure 5:**
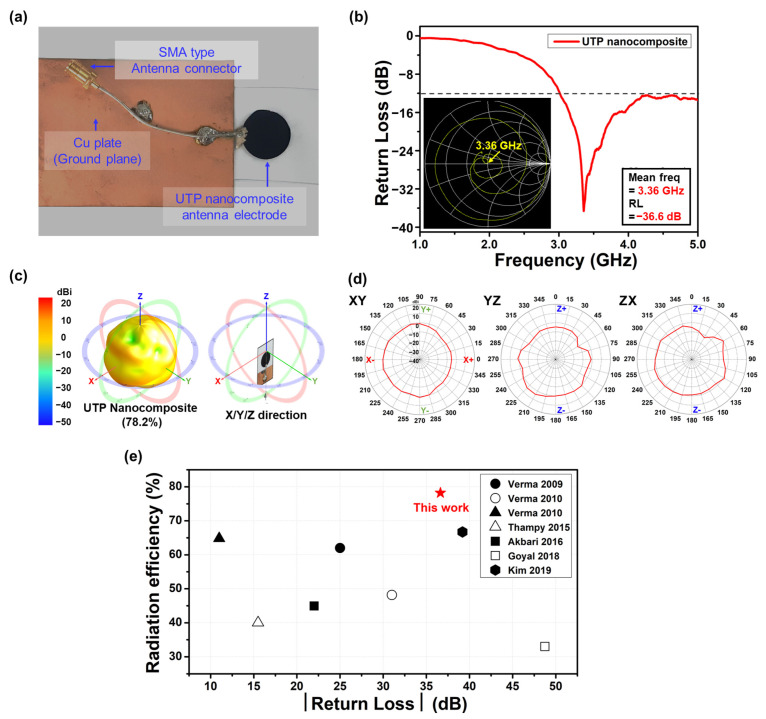
(**a**) Image of a free-standing UTP nanocomposite-based monopole patch RF antenna with a rectangular Cu ground plane. (**b**) RL curves (inset: Smith chart impedance diagram). (**c**) 3D and (**d**) 2D radiation patterns. (**e**) Comparison of RL and radiation efficiency values for various PPY-based RF antennas and graphene-based RF antennas [37,38,39,40,41,42,43].

As shown in Figure 5a, impedance matching was performed for the pelletized UTP nanocomposite when utilized as a monopole patch RF antenna. The antenna utilized a rectangular Cu plate as the ground plane and a subminiature version A connector. In general, antenna performance is evaluated based on its mean frequency and parameters such as bandwidth and RL [31]. Figure 5b shows the mean frequency and RL values of the UTP nanocomposite-based monopole patch RF antenna. The antenna characteristics are listed in Table 1. The RL value refers to the loss of power caused by impedance mismatching. To optimize power transfer and minimize reflections, reducing the impedance mismatch between the input and output is essential. Generally, it is expressed as a ratio in decibels (dB) and is evaluated as follows:

$$\mathrm{RL}\;\left(\mathrm{dB}\right)=10\;{log}_{10}\frac{P_{r}}{P_{i}}$$

where *P_r_* is reflected power and *P_i_* is incident power [32]. The inset to Figure 4b shows a typical Smith chart impedance diagram of the UTP nanocomposite-based monopole patch RF antenna [33]. The impedance point near the center of the Smith chart indicates the lowest RL value, which is −36.23 dB. It also indicates the highest transmitted power efficiency at 100%. Furthermore, the point where the RL value is at its minimum indicates the mean frequency of the antenna. The UTP nanocomposite antenna exhibited a mean frequency of 3.36 GHz; hence, it can be applied to 5G technology [34]. Bandwidth is another important factor in determining the performance of the antenna. It represents the range of frequencies (RL below −10 dB) over which the antenna can efficiently radiate or receive energy. Considering this, it is evident that the UTP nanocomposite antenna is capable of transmitting across a wide range of frequencies.

To gain further insight into the performance of the antenna, both radiation efficiency and power gain were measured. Figure 5c depicts the 3D radiation pattern of the UTP nanocomposite-based monopole patch RF antenna. Radiation efficiency is the parameter used to describe how effectively an antenna transmits and receives RF signals. It is defined as the ratio of the total power radiated by an antenna to the total input power received from the generator [35,36]. Power gain is a parameter used to indicate the directivity of the antenna. It is the measure of the strength of energy in a particular direction when referenced to isotropic radiation, which assumes equal radiation in all directions. The proposed antenna exhibited a high radiation efficiency of 78.2%.

Figure 5d shows the 2D radiation patterns, which were obtained from the 3D radiation patterns by dividing the latter into vertical and horizontal planes. Omnidirectional electromagnetic radiation was observed in the 2D radiation patterns of the UTP nanocomposite-based monopole patch RF antenna. This result indicates the ability of the proposed antenna to transmit equally in all directions. This isotropic property has the advantage of being able to receive electromagnetic waves from any direction. The concave area in the top-left corner of the YZ and ZX planes was influenced by the connection of the antenna and adapter during radiation efficiency measurements.

The radiation-efficiency values of various antennas using the conductive polymer PPY or the 2D conductive material graphene are plotted with respect to the absolute RL value in Figure 5e [37,38,39,40,41,42,43]. These values, when compared with those of the UTP nanocomposite-based monopole patch RF antenna, reveal that the proposed antenna exhibited outstanding performance, including high transmitted power and radiation efficiency. These results suggest that the highly electrically conductive and free-standing UTP nanocomposite pellet can be successfully utilized as an omnidirectional monopole patch RF antenna. Furthermore, the fact that there is no significant resistance change even after 12 weeks ensures the long-term stability of the UTP nanocomposite-based monopole patch RF antenna.

## 4. Conclusions

In conclusion, an effective and simple method was employed for the fabrication of an omnidirectional and free-standing UTP nanocomposite-based monopole patch RF antenna. The UTP nanocomposite antenna was fabricated by ball milling of U-FP, chemical oxidative polymerization of UTP nanocomposite, and mechanical pelletizing of the composite. The anatase TiO_2_ nanoparticles enhanced the conductivity and prevented the oxidation of the UTP nanocomposite. Owing to these characteristics, the UTP nanocomposite exhibited a low RL value, high radiation efficiency, and long-term stability. The UTP nanocomposite monopole patch RF antenna exhibited competitive performance when compared with those of RF antennas reported in previous studies, including those based on PPY or graphene. The UTP nanocomposite-based monopole patch RF antenna has significant potential for wireless communication applications in 5G technology.

## Figures and Tables

**Table 1:** Antenna characteristics of the UTP nanocomposite-based monopole patch antenna.

Mean Frequency (GHz)	VSWR	Absolute RL Value (dB)	Transmitted Power (%)	Peak Directivity (dBi)	Peak Gain (dBi)	Radiation Efficiency (%)
3.36	1.03	36.6	100	5.69	4.45	78.2

## Data Availability

Data are contained within the article and Supplementary Materials.

## Supplementary Materials

The following supporting information can be downloaded at: https://www.mdpi.com/article/10.3390/nano14121013/s1, Figure S1. AFM image of black phosphorus. Figure S2. (a) FE-SEM images of U-FP/PPY and (b) TiO_2_/PPY-based binary nanocomposite. Figure S3. FT-IR spectra of UTP nanocomposite.
