# Supplementary material for: Highly Conductive and Long-Term Stable Phosphorene-Based Nanocomposite for Radio-Frequency Antenna Application
Source: Nanomaterials (Basel). 2024 Jun 12;14(12):1013. doi: 10.3390/nano14121013 (PMC11206362; doi:10.3390/nano14121013)
Supplement: Supplementary file 1 [file nanomaterials-14-01013-s001.zip › nanomaterials-3030506-supplementary.pdf]

Supporting Information

Highly Conductive and Long-Term Stable Phosphorene-Based Nanocomposite for Radio-Frequency Antenna Application

Kibum Song †, Seungho Ha † and Keun-Young Shin *

Department of Materials Science and Engineering, Soongsil University, 369 Sangdo-ro, Dongjak-gu, Seoul 06978, Republic of Korea; kibumsong121@naver.com (K.S.); hsho312@naver.com (S.H.)

* Correspondence: skykek@ssu.ac.kr

† These authors contributed equally to this work.

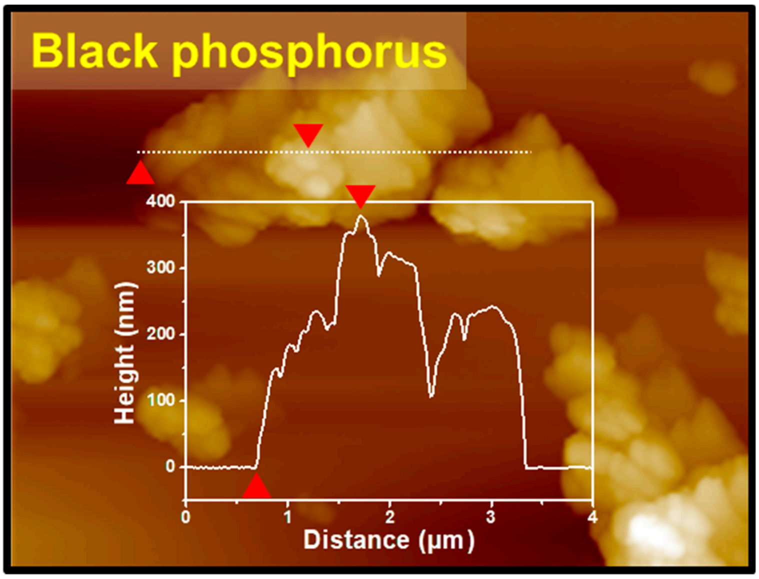

Figure S1. AFM image of black phosphorus.

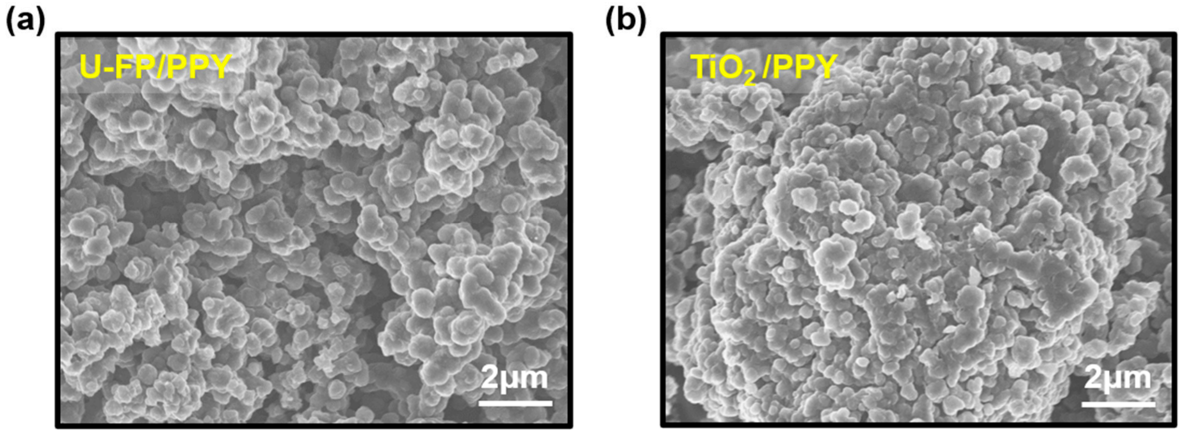

Figure S2. (a) FE-SEM images of U-FP/PPY and (b) TiO_2/PPY-based binary nanocomposite.

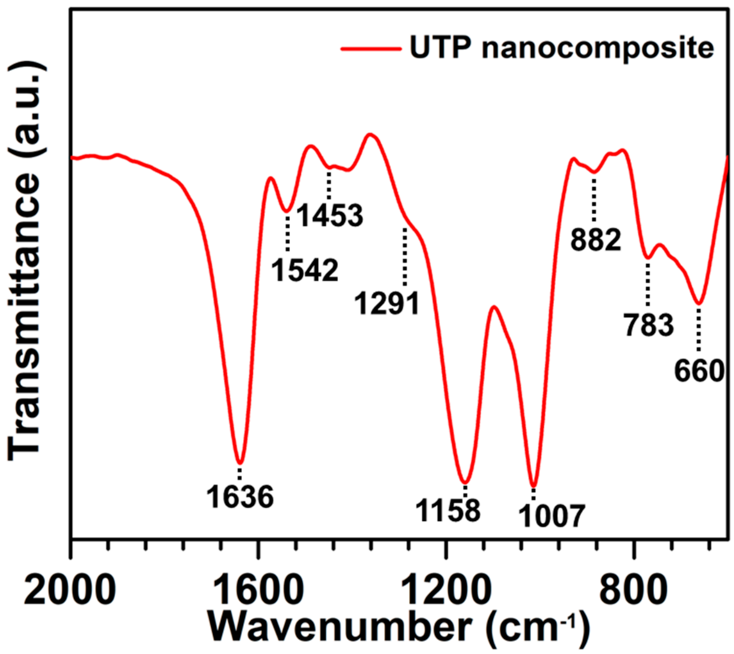

Figure S3. FT-IR spectra of UTP nanocomposite.
